# The Effectiveness of Small Group Education on Improving Antibiotic Prescribing in General Practice: A Mixed Methods Systematic Review

**DOI:** 10.3390/antibiotics15050458

**Published:** 2026-04-30

**Authors:** Kevin F. Roche, Anthony Maher, Eimear C. Morrissey, Rosie Dunne, Andrew W. Murphy, Babatunde Ayeni, Gerard J. Molloy

**Affiliations:** 1 School of Psychology, University of Galway, H91 TK33 Galway, Ireland; 2 Centre for Health Research Methodology, University of Galway, H91 TK33 Galway, Ireland; 3 Institute for Clinical Trials, University of Galway, H91 TK33 Galway, Ireland; 4 Library, University of Galway, H91 TK33 Galway, Ireland; 5 Health Research Board Primary Care Clinical Trials Network, University of Galway, H91 TK33 Galway, Ireland; 6 Midlands Training Scheme, Irish College of General Practitioners, D02 DK23 Dublin, Ireland

**Keywords:** general practice, continuing medical education, small group learning, practitioner prescribing

## Abstract

**Background/Objectives**: Reducing inappropriate use of antimicrobial agents in healthcare settings is a critical strategy to mitigate the growing threat of antimicrobial resistance. Globally, the highest consumption of antimicrobials in human healthcare originates from antibiotic prescriptions made in General Practice settings. Small group learning has long held a key role in General Practice education, characterized by active participation, common learning goals, and opportunities for reflection. This mode of delivery has been explored as a potential approach to increase appropriate antibiotic prescribing, supported by research indicating that more didactic educational interventions are unlikely to effectively improve physician prescribing behaviours. This systematic review specifically sought to synthesise the evidence on the effectiveness of small group-based, interventions in improving appropriate antibiotic prescribing behaviours in general practice. **Methods**: A mixed methods systematic review was employed. Studies were eligible if they reported on in-person, small group-based educational interventions to improve antibiotic prescribing among GPs. Full-text screening resulted in 19 eligible studies. Key characteristics, such as study design, intervention content, and outcomes, were extracted. **Results**: The 19 included studies used single and multi-modal interventions, with 68% focusing on respiratory tract infections. Common topics were patient communication (*n* = 11) and adherence to prescribing guidelines (*n* = 8). Most (*n* = 11) reported positive outcomes like reduced prescribing and were acceptable to GPs. **Conclusions**: These types of interventions can be effective in increasing the appropriate use of antibiotics in General Practice and are well received by GP participants. However, further research is required on the optimal content delivered in interventions and their associated long-term impact.

## 1. Introduction

Antimicrobial resistance (AMR) is increasingly recognised as a threat to global health [1,2] with impacts that can potentially extend beyond infectious diseases. The overconsumption, and particularly the inappropriate consumption, of antimicrobials, especially antibiotics, has been identified as a key feature in the advance of AMR globally [3]. One element that has been highlighted as particularly relevant in human health is the antimicrobial prescribing behaviours of healthcare professionals [4].

Appropriate antimicrobial prescribing refers to the judicious use of these agents only when clinically indicated. It also involves selecting the correct drug at the appropriate dosage for the designated duration. Inappropriate prescribing of antibiotics is considered to be when antibiotics are prescribed for conditions despite there being limited evidence that the antibiotics would provide any benefit [5]. The AMR focus of this review is on the increasing bacterial resistance arising from the overuse of antibiotics, often through inappropriate prescribing [6] in Primary Care settings, as the majority of human antibiotic consumption internationally originates in this setting [7,8]. Despite the increase in the promotion of appropriate antibiotic prescribing behaviours in General Practice [9], inappropriate prescribing still occurs due to a number of different factors [10], and General Practitioners’ (GPs) antibiotic prescribing practices, once established, tend to remain consistent [11]. As the focus of this review is on interventions targeting GPs, we are referring to the settings as General Practice as opposed to Primary Care, which could also potentially include non-GP prescribers such as Nurses and Pharmacists.

Small group learning in General Practice has long been a cornerstone of post-graduate and continuing General Practice education [12], with proven benefits for participants [13]. For our working definition of small group learning we are following the characteristics laid out by Crosby (1996), where the group must allow for active participation, there must be a common learning goal and there should be an opportunity for reflection [14]. This definition of small group learning is also supported by other research that identifies the key characteristics as being to identify gaps between current practice and best available evidence, to advocate for changes in patient care and to encourage reflection on current individual practice [15]. The use of this mode of delivery to improve appropriate antibiotic prescribing in General Practice is supported by a 2005 Cochrane review exploring interventions to improve antibiotic prescribing in ambulatory care, which found that the use of more didactic educational interventions, such as lectures, were unlikely to improve the antibiotic prescribing of physicians in ambulatory care [16].

This review focuses on small group learning, a format that has been effectively used to integrate updated clinical knowledge for patient care in General Practice settings [12]. Small group learning, as a format, has also been used to address instances of clinical uncertainty encountered by GPs in their daily practice [17]. GPs can experience clinical uncertainty in the prescribing of antibiotics, for example, when parents of children presenting to the GP offer candidate diagnoses, which can be interpreted as advocating for antibiotics [18].

The present review aims to systematically evaluate and synthesise all the relevant and available evidence on the efficacy and potential effectiveness of small group-based educational interventions delivered in person to GPs that aim to improve antibiotic prescribing behaviours. The review will also review the topics, format and content of the studies, where reported. The aim of this review is to inform the delivery and content of future training for General Practitioners on the topic of antibiotic prescribing.

## 2. Methods

### 2.1. Aim

The aim of this review was to systematically review the scientific literature on the use of small group-based in-person educational interventions designed to improve antibiotic prescribing behaviours in General Practice. Specifically, this review reports on the following questions: What is the efficacy and potential effectiveness of small group-based in-person educational interventions designed to improve antibiotic prescribing behaviours in General Practice? What are the components of these interventions? What, if any, are the insights of intervention participants?

### 2.2. Design

This systematic review was conducted following the Preferred Reporting Items for Systematic Reviews and Meta-analysis (PRISMA) 2020 guidelines [19] and followed a predefined protocol [20]. The PRISMA checklist is available in the Supplementary Materials. The review protocol was registered in the International Prospective Register of Systematic Reviews (PROSPERO) CRD42024512491.

### 2.3. Search Methods

The search strategy was developed between the lead author (KR) and an academic research librarian (RD). Studies were identified by searching the electronic databases Embase, Medline (OVID), Cinahl, Cochrane Central, PsycINFO (OVID) and PubMed. The searches were run during February 2025 and were updated in April 2026, and no new studies were identified. Studies included were from database inception onwards and were limited to full-text studies reported in the English language. Searches were limited to studies reported in English due to time and resource constraints. The search strategy used both medical subject headings (MeSH) and free-text terms. The search strategy was modified for each of the databases. The search strategy for Medline (OVID) is attached in the Supplementary Data. The search strategies for the other databases are available online at DOI 10.17605/OSF.IO/ET2GX.

### 2.4. Search Outcomes

**Figure 1:**
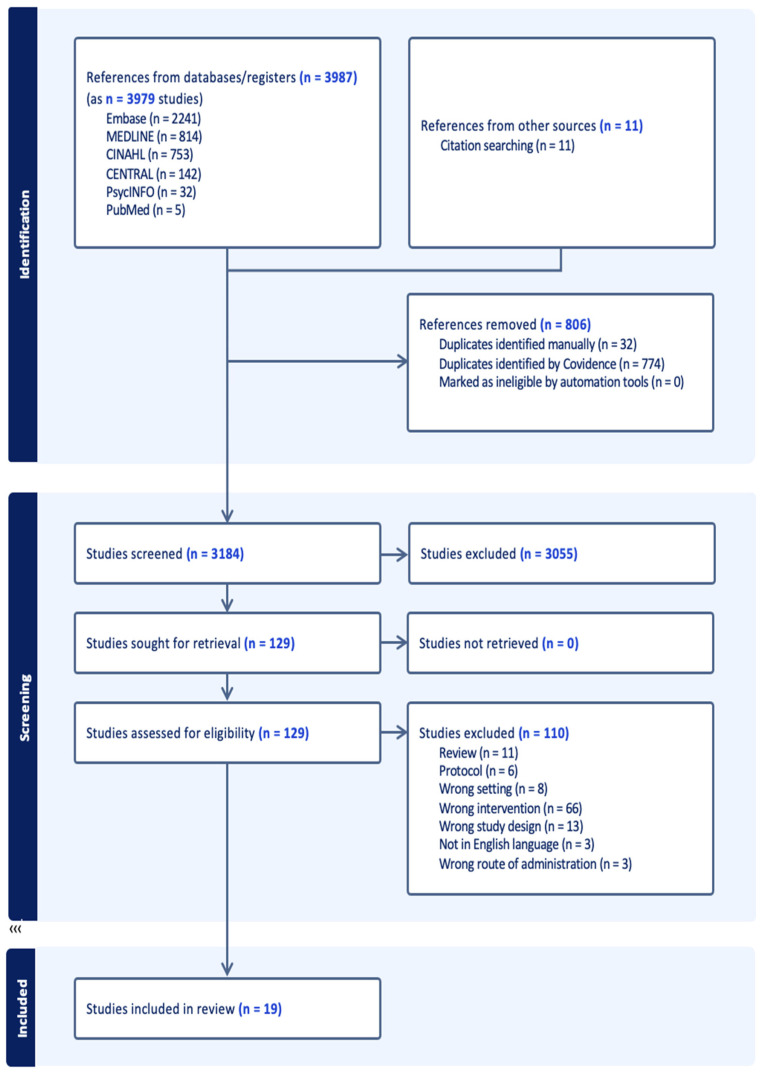
Prisma flowchart.

The results from all searches (3987 references) were imported into COVIDENCE. After screening for duplicates, 3184 papers were double-screened at title and abstract level with the one author (KR) screening 100% and two other members of the research team (AMR and BA) screening 50% each. A total of 128 studies were identified from the initial screening and brought forward to full-text screening. For studies identified at this stage of screening, where the original studies did not contain enough information to decide whether to include or exclude and where relevant data was contained in other published works, e.g., protocols, the additional works were reviewed before a decision was made. A full list of the additional studies consulted for the included studies id listed in the Supplementary Data. After full-text screening, 19 studies were identified over 27 published papers. The PRISMA flowchart is presented in Figure 1. Full-text screening was carried out by KR, with a random 20% of the articles screened by a second author (AMR). Any disagreement regarding inclusion or exclusion was resolved through discussion. The studies eligible for inclusion were those that:

1. considered the use of in-person small group-based educational interventions, either as part of broader interventions or as stand-alone interventions, to optimise antibiotic prescribing in General Practice settings.
2. focused on General Practitioners (GPs) or GP Registrars (qualified doctors engaged in General Practice post-graduate training).
3. were published empirical studies

### 2.5. Quality of Studies Appraisal

The quality of the included studies was appraised using the Mixed Methods Appraisal Tool (MMAT—version 2018) [21]. The MMAT is used to appraise the methodological quality of quantitative, qualitative or mixed methods studies and has been used previously in mixed methods systematic reviews [22,23,24]. Each of the 19 included studies was appraised by KR, with a random selection of 4 studies (~20%) further appraised by EM. As there was high consistency in the appraisal of the selected studies and there has previously been reviewer consistency [25,26], this was considered sufficient. Following the recommendations of the MMAT [21,27], no studies were excluded from the review. The MMAT for the included studies is reported in the Supplementary Data.

### 2.6. Data Extraction

Data extraction on the 19 studies was carried out by one team member (KR) using a pre-defined data extraction sheet. A random selection of 20% of the studies were double-screened by AM to independently verify the data extraction. The verification of the data extraction was consistent and there has been previous reviewer coherence [25]; thus, the necessary requirements for data extraction were considered met. The data extraction fields included: author(s); year of publication; country of origin; study design; population; specific disease focus (if any); outcomes reported; intervention components; workshop content and additional notes, comments or insights.

### 2.7. Synthesis

Data synthesis was carried out using a narrative synthesis approach, following the steps outlined by Popay et al. [28]. At the preliminary analysis stage we identified if the included studies were single modality interventions or part of larger multi-modal interventions and this data is included in Table 1. The primary outcomes of the reported studies are presented in Table 2. The second stage of the approach exploring the relationships within and between studies involved developing descriptive themes of the content of the workshop components of the included studies and the feedback from participants, where possible. The robustness of the synthesis was then appraised, and we developed our theoretical model on the use of group-based in-person educational interventions to address inappropriate antibiotic prescribing in General Practice settings.

## 3. Results

### 3.1. Summary of Included Studies

There are 19 studies included in this mixed methods systematic review. The specific details, year, location, study design, main disease focus, sample and outcomes measured are presented in Table 1.

### 3.2. Intervention Characteristics

The included studies were stratified at two levels. The first level focused on intervention characteristics and what, if any, modalities in addition to the small group format were used in the interventions. The second level focused on the content of the small group meeting components of the interventions. The included studies contained a mix of single modality and multiple modality interventions. Six studies used an educational intervention, in the small group format, exclusively [29,37,38,39,40,43]. Of the 15 studies that reported outcomes of the interventions, 11 reported positive outcomes [27,29,30,32,34,36,37,39,41,42,43] and one reported a positive outcome for one aspect where there was a reduction in the prescription of antibiotics for bronchitis/bronchiolitis; however, there was no significant reduction in antibiotic prescribing for upper respiratory tract infections [31]. There was no discernible difference between whether single modality or multiple modality interventions led to positive outcomes, with four of the 11 studies reporting positive outcomes being single modality [29,37,39,43] and one single modality study reporting no reduction in antibiotic prescribing rates of participants [40]. A more detailed description of the study characteristics is provided in Supplement Table S3.

### 3.3. Narrative Synthesis

From the 19 included studies we identified that there were two main themes addressed in the educational workshop component of the interventions.

1. Patient communication strategies [25,26,27,28,30,31,35,38,40,41,42];
2. Adherence to guidelines on antibiotic prescribing [30,31,34,35,36,39,40,42].

### 3.4. Patient Communication

With 11 of the 19 included studies incorporating material or content focusing on patient communication around antibiotic prescribing, we found that this was the main topic considered in the educational workshops included in this review. All 11 studies [25,26,27,28,30,31,35,38,40,41,42] incorporating aspects of patient communication had respiratory tract infections (RTIs) as the disease focus. Regarding communicating specific aspects of antibiotic use to patients, two of the studies emphasised communicating the risks and benefits of using antibiotics to deal with RTIs [26,27]. Two studies concentrated on communication skills to manage and modify patients’ beliefs and/or concerns about the use of antibiotics in managing RTIs [25,42], with one [42] also exploring the use of communicating about the natural course of symptoms, self-medication and any red flag symptoms to be aware of. Communication strategies to improve patient participation in the decision-making process to use antibiotics for RTIs was addressed in two of the studies [26,40]. Four studies used specific communication skills training; one described the use of active listening techniques, responding to emotional cues and the tailoring of information given to patients about antibiotic use [25]; and another of the studies discussing specific communication skills training emphasised that when addressing patients who present with RTIs, the consultation techniques of the GP registrars (the population of interest in the study) should reflect the biopsychosocial complexity of the presentation rather than the biological complexity [31]. The acceptability of the use of context-bound training in communication skills where the training focused on using communication skills to resolve the everyday problems of GPs was carried out by one study [38]. One study used a specific communication skills training tool [48] in conjunction with providing information about the associated evidence regarding the treatment of RTIs [41]. One of the 11 studies focusing on patient communication listed communication as one strategy to avoid antibiotic prescribing [35]. Another study reported the use of communication training as part of the workshop component of the intervention but did not report other information, although as a multi-modal intervention it listed culturally sensitive communication and effective skills training as elements of the pre-workshop e-learning modules [28]. Only one of the 11 studies discussed patient communication in terms of managing patient expectations and perceptions around using antibiotics to manage RTIs [31].

### 3.5. Following Guidelines on Antibiotic Prescribing

Eight out of the 19 included studies had following appropriate guidelines or following recommendations as part of the content of the educational workshop [30,31,34,35,36,39,40,42]. One study reported using a combination of national and international (World Health Organisation) antibiotic prescribing guidelines [39]. One study [35] did not report where the prescribing recommendations used in the intervention were drawn from. Two studies reported developing their own recommendations/guidelines for the intervention [34,36]. Four studies reported using national prescribing guidelines [30,31,40,42], although these were only based in two countries (Australia and The Netherlands).

### 3.6. Feedback from Participants on Educational Workshops

Seven [26,28,29,32,33,35,38] of the 19 included studies reported on feedback from the participants of the educational workshop components of the interventions. Two of the studies reported the feedback in separate papers outside of the main studies; these were McNulty (2018) [36] and Lundborg (2011) [33], with the feedback being reported in Jones (2018) [49] and Lundborg (1999) [50], respectively. One study [38] reported solely qualitative data, and another [45] reported both qualitative and quantitative data. All feedback on the workshop component of the interventions was positive. Petruschke [35] reported that of all the intervention components the educational workshop was rated highest by the intervention participants. In the study that reported qualitative feedback [38] the participants identified that the relevance of the workshop to their daily practice was a factor in the positive outlook toward the workshop. The use of case-based scenarios and the opportunity to discuss these with other participants was identified as being the most useful aspect of the workshops. One of the included studies [28] reported that the content of the workshop targeting the patient population was of greater benefit to the participants than the content related to antibiotic prescribing. This was echoed in another of the studies [45] where the introductory information of the workshop, where the topic of AMR was addressed, was criticised by the participants as repeating well-known information.

## 4. Discussion

This systematic review is, to our knowledge, the first to review the use of in- person educational meetings/workshops to improve antibiotic prescribing in General Practice settings. The use of this format in addressing antibiotic prescribing in General Practice settings seems to be effective in terms of its acceptability by participants irrespective of being a single modal intervention or as part of a broader multi-modal intervention. The majority of the interventions focused on the use of antibiotics with respiratory tract infections (RTIs). This is in line with previous work stating that RTIs are the most frequent reason for non-routine consultations in General Practice [51] and also a 2014 systematic review [52] that found 70% of all educational interventions addressing antibiotic use in Primary Care focused on respiratory infections. The evidence indicating that an educational workshop component was rated the highest when part of a multi-modal intervention is supported by other research [53] as it allows GPs to discuss and compare their own antibiotic prescribing behaviours with their peers.

We found that the main topics considered in the educational meeting component were patient communication and following prescribing guidelines for the use of antibiotics. The use of patient communication skills to address antibiotic prescribing in General Practice has also previously shown to be an effective component in other types of interventions. A 2009 study based in The Netherlands [54] found that GPs who had received advanced training in patient communication skills prescribed antibiotics to 27% of patients in comparison to those who had not received the training, who prescribed antibiotics to 54% of their patients.

There were a number of strengths in this review. To our knowledge, this is the first systematic review to focus exclusively on in-person small-group educational interventions for antibiotic prescribing in General Practice. Additionally, using a mixed methods approach allowed us to explore the findings in depth and to develop the themes of the intervention content as well as identifying that small group education delivery was acceptable to participants regardless of whether it was a single modal intervention or part of a larger multi-modal intervention.

Regardless of the strengths, there are some limitations in this review that must be acknowledged, including the heterogeneity of the included studies in terms of their PICO characteristics, which did not allow for a meta-analysis to be conducted. While this review also identified that there were no discernible differences between whether single modality or multiple modality interventions led to positive outcomes, it is important to note that due to the lack of formal comparative analysis, because of the heterogeneity of the included studies, this finding should be interpreted cautiously. The methodological quality was appraised using the Mixed Methods Appraisal Tool (MMAT); however, the heterogeneity of included study designs limited the extent to which quality ratings could be directly incorporated into the synthesis. In particular, randomised and non-randomised studies contributed primarily to conclusions regarding effectiveness, whereas feasibility and acceptability studies provided contextual insights into implementation. Future work could apply approaches such as GRADE to formally assess certainty of evidence for effectiveness outcomes. The development of a core outcome set for antibiotic stewardship in General Practice, similar to dentistry [55], would enable significant intervention design and allow for comprehensive comparison of interventions. The development of a core outcome set would also add precision to the reported outcomes of antibiotic prescribing interventions in General Practice settings and allow for the development of a cumulative evidence base. Due to resource constraints only studies published in English were included; therefore, there is a language bias and publication bias cannot be excluded.

### Implications

Our finding that the content of the small group-based educational interventions was mainly limited to patient communication and adherence to guidelines highlights that these topics are well covered in this type of educational intervention. Future curriculum design of interventions that use this format could incorporate other topics that have been found to have an impact on physician prescribing of antibiotics [51], such as clinical experience, diagnostic uncertainty, patient volume and managing time pressure. Small group learning has been consistently associated with important psychological and educational benefits in medical and healthcare training, particularly through its promotion of active, socially mediated cognition [56]. In continuing medical education, group-based approaches additionally integrate personal and professional experiences, enabling reflective dialogue and emotional insight (e.g., Balint-style groups), which can influence attitudes and empathy in clinical care [57]. Small group learning settings are ideally positioned for addressing these intrapersonal factors that can influence antibiotic prescribing in General Practice settings. Considering that health system-related factors also have an impact on physician prescribing [58], it is important to consider the context that any intervention will be designed for and to tailor any intervention not just to the professional population but also the systems within which the intervention will be carried out.

## 5. Conclusions

This review explored the use of in-person small group-based educational interventions to improve antibiotic prescribing in General Practice settings. The aim of this review was to inform the delivery and content of future training for General Practitioners on the topic of antibiotic prescribing. The findings show that using this mode for delivering an intervention to GPs either singly or as part of multi-modal interventions can be efficacious in increasing the appropriate use of antibiotics in General Practice and as a modality is well received by GP participants in continuing medical education settings. This is an important factor as evidence from healthcare training and behaviour change interventions indicates that acceptability is an important implementation construct associated with participant adherence and intervention fidelity [59,60]. Therefore, the inclusion of these strategies can help play a part in addressing the complex challenge of AMR. By identifying that the contents of these interventions mainly focus on interpersonal factors we have identified that there is scope for further research on the use of intrapersonal factors in designing future interventions to improve antibiotic prescribing in General Practice. Larger long-term effectiveness and cost-effectiveness analyses are needed to establish the appropriate resourcing and prominence of this topic in an ever-expanding landscape of GP continuing medical education.

## Figures and Tables

**Table 1:** Characteristics of included studies (PCO of PICO).

Author, Year, Country	Study Design/Methods	Participants	Disease Focus	Primary Outcome Measurement	Secondary Outcomes	Comparator	Workshop Format
Briel et al. (2006) [29], Switzerland	Randomised controlled trial	GPs, 45 (15, control, 15, limited intervention, 15, full intervention)	Acute respiratory tract infections	Antibiotic prescription rates as reported by Pharmacists	Patient satisfaction and enablement, re-consultation rates, days with restrictions, days off work	Limited intervention Usual care	Six-hour seminar
LeBlanc et al. (2011) [30], Canada	Pilot clustered randomised trial	Family Physicians, 39 (18, intervention, 21, control) Patients, 544	Acute respiratory infections	Feasibility and acceptability of the intervention	Reported elsewhere	Usual care (control group received delayed exposure)	Three 3 h workshops delivered over a 4–6-month period
Légaré et al. (2012) [31], Canada	Clustered randomised trial	Family Physicians, 149 (77, intervention, 72, control) Patients, 359 (181, intervention, 178, control)	Acute respiratory infections	Proportion of patients who decided to use antibiotics immediately after consultation	Decisional conflict, shared decision making, quality of decision	Usual care	2 h seminar
Lescure et al. (2024) [32], The Netherlands	Non-randomised controlled before–after study	GPs, 135 (25, intervention, 110, control)	Respiratory tract infections (RTIs)	Absolute number of prescribed antibiotic courses indicated for RTIs per GP	Mean number of all prescribed antibiotic courses per GP	Usual care	3 h training session
Lundborg et al. (1999) [33], Sweden	Randomised controlled trial (2 parallel interventions)	GPs, 204 (104, antibiotics intervention, 100, asthma intervention)	Urinary tract infections and asthma	Prescribing indicators	Knowledge and attitudes toward asthma and UTIs, knowledge, attitudes and judgements on simulated cases, evaluation of educational model	Usual care	Two educational sessions (averaging 1.4 h each in duration)
Magin et al. (2016) [34], Australia	Pre- and post-intervention comparison of intention to prescribe	GP Registrars (Trainees), 89 GP Training Supervisors, 124	Respiratory tract infections	Comparison of Registrars’ pre- and post-intervention intention to prescribe antibiotics	Comparison of supervisors’ pre- and post-intervention intention to prescribe antibiotics	n/a	90 min educational session
Magin et al. (2018) [35], Australia	Pragmatic prospective non-randomised controlled trial	GP Registrars (Trainees) (217, intervention, 311, control)	Upper respiratory tract infections (URTIs) and acute bronchitis/bronchiolitis	Prescription/non-prescription of antibiotics for presentations with URTI and acute bronchitis/bronchiolitis	n/a	Usual education provision	90 min educational session
McNulty et al. (2018) [36], United Kingdom	Pragmatic randomised controlled trial	GP Surgeries, 150 (73, intervention, 75, control) (166 GPs, intervention)	General antibiotic use	Total oral antibiotics dispensed per 1000 practice patients	Workshop uptake, dispensing of antibiotics typically prescribed for RTIs, UTIs and broad-spectrum antibiotics	Usual care	1 h workshop
Morrison et al. (2005) [37], Scotland	Assessment of the feasibility and acceptability of an intervention	GPs, 24	General antibiotic use	Assessment of the feasibility and acceptability of a problem-based, peer-facilitated educational workshop about antibiotic prescribing	n/a	n/a	2.5 h workshop
Perez-Cuevas et al. (1996) [38], Mexico	Quasi-experimental design	Family Physicians, 119 (65, intervention, 54, control)	Rhinopharyngitis	Post-intervention evaluation of short-term impact and long-term effect of intervention on appropriateness of patient treatment	n/a	Usual care	Five daily 2 h sessions over 1 week
Petruschke et al. (2021) [39], Germany	Evaluation of an intervention	GPs, 107	Acute respiratory tract infections	Evaluation of the intervention	n/a	n/a	2 h training session
Reyes-Morales et al. (2009) [40], Mexico	Non-randomised pre–post intervention	Family Physicians, 106 (48, intervention, 58, control)	Acute respiratory infections (ARIs)	Appropriate case management of ARIs	n/a	Usual care	Five 1 h training sessions
Richards et al. (2003) [41], New Zealand	Retrospective analysis of a controlled trial	GPs, 230 (52, intervention, 178, control)	Prescribing in General Practice where gaps were identified between ideal and actual prescribing, including antibiotics	Targeted prescribing data for 12 months before and 24 months after each of four education sessions	n/a	Usual care	Not reported
Rollnick et al. (2002) [42], United Kingdom	Acceptability of a training method	GPs 3 Groups (Group 1 = 5, Group 2 = 6, Group 3 not reported)	Acute respiratory infections	Acceptability of the intervention	n/a	n/a	Three weekly seminars of 45 min duration
Sharma et al. (2002) [43], India	Prospective randomised controlled pre–post intervention and qualitative interviews	Dispensary-Based Physicians Number not reported	Acute upper respiratory infection (ARI) and diarrhoea	Average antibiotic prescribing rates between control and intervention groups	n/a	Usual care	Day long interactional workshop
Smeets et al. (2009) [44], The Netherlands	Controlled before and after study	GPs, 258 (131, intervention, 127, control)	Respiratory tract infections	Number of antibiotic prescriptions per 1000 patients	Number of second choice antibiotics	Usual care	Not reported
Strumann et al. (2020) [45], Germany	Controlled trial	Primary Care Physicians, 17 (17, intervention, used entropy balancing to match with treatment cases from observational data)	Upper respiratory tract infections	Binary choice whether an antibiotic was prescribed for a URTI case	n/a	Usual care	Two × 2.25 h training
Welschen et al. (2004) [46], The Netherlands	Randomised controlled trial	GPs, 89 (42, intervention, 47, control)	Respiratory tract symptoms	Antibiotic prescription rates for acute symptoms of the respiratory tract and patients’ satisfaction	Claims data between 2000–2002, volume of antibiotic prescribing before and after intervention	Usual care	Not reported
Wilf-Miron et al. (2012) [47], Israel	Between-group comparison	GPs, 83 (11, intervention, 72, control)	General antibiotic use	Pre–post intervention prescribing rates	n/a	Usual care	Four structured meetings of 4 h each, 2 months apart

**Table 2:** Primary outcomes.

Author, Year, Country	Study Design/Methods	Primary Outcome Measurement	Effect Estimate/Results
Briel et al. (2006) [29], Switzerland	Randomised controlled trial	Antibiotic prescription rates as reported by Pharmacists	No significant differences in Pharmacist-reported uptake of antibiotic prescriptions between control and intervention; 0.86 (95% CI 0.40–1.93) ^1^
LeBlanc et al. (2011) [30], Canada	Pilot clustered randomised trial	Feasibility and acceptability of the intervention	Overall level of satisfaction with workshops reported at 94%
Légaré et al. (2012) [31], Canada	Clustered randomised trial	Proportion of patients who decided to use antibiotics immediately after consultation	25% difference between intervention and control patients in decision to use antibiotics; 0.48 ^2^ (95% CI 0.34 to 0.68)
Lescure et al. (2024) [32], The Netherlands	Non-randomised controlled before–after study	Absolute number of prescribed antibiotic courses indicated for RTIs per GP	No significant difference in mean number of prescribed antibiotics between control and intervention; −0.9 (95% CI −28.2–37.1) ^3^
Lundborg et al. (1999) [33], Sweden	Randomised controlled trial (2 parallel interventions)	Adherence to prescribing indicators for UTIs	Intervention showed a significant effect in desired direction, from 52% to 70%; 6.85 (95% *p*< 0.001) ^4^
Magin et al. (2016) [34], Australia	Pre- and post-intervention comparison of intention to prescribe	Comparison of pre- and post-intervention intention to prescribe antibiotics	12.0–24.0% range of absolute reduction in intention to prescribe antibiotics in 4/5 vignettes
Magin et al. (2018) [35], Australia	Pragmatic prospective non-randomised controlled trial	Prescription/non-prescription of antibiotics for presentations with URTI and acute bronchitis/bronchiolitis	No significant reduction in antibiotic prescribing for URTIs 15.8% (95% CI 4.2–27.5%) reduction in prescribing for bronchitis/bronchiolitis ^5^
McNulty et al. (2018) [36], United Kingdom	Pragmatic randomised controlled trial	Total oral antibiotics dispensed per 1000 practice patients	6.1% (95% CI 0.2–11.7%) ^6^ estimated lower total antibiotic prescribing
Morrison et al. (2005) [37], Scotland	Assessment of the feasibility and acceptability of an intervention	Assessment of the feasibility and acceptability of a problem-based, peer-facilitated educational workshop about antibiotic prescribing	Format of the intervention was highly acceptable to participants
Perez-Cuevas et al. (1996) [38], Mexico	Quasi-experimental design	Post intervention evaluation of short-term impact and long-term effect of intervention on appropriateness of patient treatment in two health systems	40% of participants improved their prescribing practices post-intervention, with this change remaining in 27.5% 18 months post-intervention period. Intervention resulted in a reduction in antibiotic prescriptions, from 85.2% to 48.1%, for patients in one health system and from 68.8% to 49.1% in another
Petruschke et al. (2021) [39], Germany	Evaluation of an intervention	Evaluation of the intervention	GP training on rational antibiotic prescribing rated the highest of the multiple intervention tools
Reyes-Morales et al. (2009) [40], Mexico	Non-randomised pre–post intervention	Appropriate case management of ARIs	19.6% difference in mean proportion of improvement compared to baseline evaluation (95% CI 11.2–28.0) ^7^
Richards et al. (2003) [41], New Zealand	Retrospective analysis of a controlled trial to promote rational prescribing	Targeted prescribing data for 12 months before and 24 months after each of four education sessions focusing on 8 key messages	Effect size ^8^ ranged from 7% to 40% over eight messages; mean effect size of 1.20 (20%) for all messages and 1.27 (27%) where effect was seen. Greatest change reported for messages relating to short-term prescriptions such as antibiotics
Rollnick et al. (2002) [42], United Kingdom	Acceptability of a training method	Acceptability of the intervention	Intervention was acceptable to participants
Sharma et al. (2002) [43], India	Prospective randomised controlled pre–post intervention and qualitative interviews	Average antibiotic prescribing rates between control and intervention groups	Significant reduction in use of antibiotics in participants post-intervention: 64.6% (95% CI; OR ^9^ 0.84) to 51.3% (95% CI; OR ^9^ 1.0) compared to 60.53% (95% CI 0.62–1.14 OR ^9^ 0.84) to 63.9% (95% CI 1.24–2.28 OR ^9^ 1.68) in control group
Smeets et al. (2009) [44], The Netherlands	Controlled before and after study	Number of antibiotic prescriptions per 1000 patients	No significant difference between control and intervention groups after intervention
Strumann et al. (2020) [45], Germany	Controlled trial	Binary choice whether an antibiotic was prescribed for a URTI case	−6.5% difference between control and intervention groups (95% CI −10.7% to −2.3%) ^10^
Welschen et al. (2004) [46], The Netherlands	Clustered randomised controlled Trial	Antibiotic prescription rates for acute symptoms of the respiratory tract and patients’ satisfaction	Mean difference in change between control and intervention group prescription rates—12% (95% CI −18.9% to −4.0%) ^11^
Wilf-Miron et al. (2012) [47], Israel	Between-group comparison	Pre–post intervention prescribing rates	Significant decline (0.05) in antibiotic prescribing rates in intervention group vs. comparison group ^12^

^1^ Adjusted odds ratio (95% CI). ^2^ Adjusted relative risk ratio (95% CI). ^3^ Adjusted post-test (95% CI). ^4^ D.f. = 32, critical t-value = 2.03 for 0.05 level 2-sided test. ^5^ Adjusted absolute reduction (95% CI). ^6^ Causal effect (CACE) analysis. ^7^ Differences in differences model, adjusted for cluster sampling. ^8^ Standardised prescribing ratio. ^9^ Odds ratio. ^10^ Difference in difference estimate. ^11^ Unpaired *t*-test. ^12^ Paired *t*-test analysis (*p* value ≤ 0.001).

## Data Availability

The search strings for all the databases are available on the OSF platform at DOI 10.17605/OSF.IO/ET2GX.

## Supplementary Materials

The following supporting information can be downloaded at: https://www.mdpi.com/article/10.3390/antibiotics15050458/s1. Table S1. PRISMA 2020 Checklist. Table S2. Study characteristics. Table S3. MMAT quality evaluation of the studies included in the mixed-method systematic review.
