# Supplementary material for: The Effectiveness of Small Group Education on Improving Antibiotic Prescribing in General Practice: A Mixed Methods Systematic Review
Source: Antibiotics (Basel). 2026 Apr 30;15(5):458. doi: 10.3390/antibiotics15050458 (PMC13203494; doi:10.3390/antibiotics15050458)
Supplement: Supplementary file 1 [file antibiotics-15-00458-s001.zip › antibiotics-4257553-supplementary.pdf]

Supplementary Data

Medline (OVID) search strategy

1 (antibiotic* or anti biotic* or antimicrob* or anti microb* or antibacteria* or anti bacteria*).mp.
2 exp Anti-bacterial agents/
3 exp general practice/ or exp family practice/
4 (primary care or primary health care or primary healthcare).mp.
5 Physicians/ or General Practitioners/ or exp Pediatricians/ or Physicians, Family/ or Physicians, Primary Care/
6 general pract*.mp.
7 Family practi*.mp.
8 GP*.mp.
9 rural Practi*.mp.
10 family medicine practi*.mp.
11 (intervention* or program* or health promotion* or education* or educational outreach* or training* or academic detailing* or educational meeting* or workshop or communication skill* or guideline* or group meeting* or decision support* or poster* or leaflet* or flyer* or incentive* or regulation* or regulatory or reminder* or consultation* or Medication review* or medication reconciliation* or drug review or stewardship or multiprong* or strategy or single or multicomponent* or multi component* or multiple or multifaceted or multidisciplinary or multi-disciplinary or physician aid or physician-aid or collaborative or collaboration or counselling or collaborative or shared).mp.
12 exp Drug Prescriptions/ or exp Practice Patterns, Physicians'/ or exp Inappropriate Prescribing/
13 (reduce or reduced or reduction or reducing or increase or increasing or increased or change or changing or changed or optimi$e or optimi$ing or optimi$ed or optimi$ation or effect* or effective or effectiveness or influence or influencing or influenced or impact* or feasible).mp.
14 (prescrib* or prescription* or practice* or practising or dispens* or stewardship or Antibiotic therapy or Antibiotic treatment or antibiotic prescribing or pattern* or behavior or behaviour).mp.
15 exp Education/
16 (graduate* adj5 education).mp.
17 (continuing adj5 education).mp.
18 (professional adj5 education).mp.
19 (professional adj5 training).mp.
20 1 or 2
21 3 or 4 or 5 or 6 or 7 or 8 or 9 or 10
22 11 or 12 or 13 or 14
23 15 or 16 or 17 or 18 or 19
24 20 and 21 and 22 and 23

Table S1. PRISMA 2020 Checklist

Section and Topic	Item #	Checklist item	Location where item is reported
**TITLE**			
Title	1	Identify the report as a systematic review.	Title
**ABSTRACT**			
Abstract	2	See the PRISMA 2020 for Abstracts checklist.	Abstract
**INTRODUCTION**			
Rationale	3	Describe the rationale for the review in the context of existing knowledge.	Introduction (2-3)
Objectives	4	Provide an explicit statement of the objective(s) or question(s) the review addresses.	Introduction (3)
**METHODS**			
Eligibility criteria	5	Specify the inclusion and exclusion criteria for the review and how studies were grouped for the syntheses.	Methods (5)
Information sources	6	Specify all databases, registers, websites, organisations, reference lists and other sources searched or consulted to identify studies. Specify the date when each source was last searched or consulted.	Methods (4)
Search strategy	7	Present the full search strategies for all databases, registers and websites, including any filters and limits used.	Example provided
Selection process	8	Specify the methods used to decide whether a study met the inclusion criteria of the review, including how many reviewers screened each record and each report retrieved, whether they worked independently, and if applicable, details of automation tools used in the process.	Data extraction (7)
Data collection process	9	Specify the methods used to collect data from reports, including how many reviewers collected data from each report, whether they worked independently, any processes for obtaining or confirming data from study investigators, and if applicable, details of automation tools used in the process.	Data extraction (7)
Data items	10a	List and define all outcomes for which data were sought. Specify whether all results that were compatible with each outcome domain in each study were sought (e.g. for all measures, time points, analyses), and if not, the methods used to decide which results to collect.	Data extraction (7)
	10b	List and define all other variables for which data were sought (e.g. participant and intervention characteristics, funding sources). Describe any assumptions made about any missing or unclear information.	n/a
Study risk of bias assessment	11	Specify the methods used to assess risk of bias in the included studies, including details of the tool(s) used, how many reviewers assessed each study and whether they worked independently, and if applicable, details of automation tools used in the process.	Quality of studies appraisal (7)
Effect measures	12	Specify for each outcome the effect measure(s) (e.g. risk ratio, mean difference) used in the synthesis or presentation of results.	Page 17
Synthesis methods	13a	Describe the processes used to decide which studies were eligible for each synthesis (e.g. tabulating the study intervention characteristics and comparing against the planned groups for each synthesis (item #5)).	Synthesis (18)
	13b	Describe any methods required to prepare the data for presentation or synthesis, such as handling of missing summary statistics, or data	Search

Section and Topic	Item #	Checklist item	Location where item is reported
		conversions.	outcomes (5)
	13c	Describe any methods used to tabulate or visually display results of individual studies and syntheses.	Data extraction (7)
	13d	Describe any methods used to synthesize results and provide a rationale for the choice(s). If meta-analysis was performed, describe the model(s), method(s) to identify the presence and extent of statistical heterogeneity, and software package(s) used.	Synthesis (18)
	13e	Describe any methods used to explore possible causes of heterogeneity among study results (e.g. subgroup analysis, meta-regression).	n/a
	13f	Describe any sensitivity analyses conducted to assess robustness of the synthesized results.	n/a
Reporting bias assessment	14	Describe any methods used to assess risk of bias due to missing results in a synthesis (arising from reporting biases).	n/a
Certainty assessment	15	Describe any methods used to assess certainty (or confidence) in the body of evidence for an outcome.	n/a
**RESULTS**			
Study selection	16a	Describe the results of the search and selection process, from the number of records identified in the search to the number of studies included in the review, ideally using a flow diagram.	PRISMA chart
	16b	Cite studies that might appear to meet the inclusion criteria, but which were excluded, and explain why they were excluded.	Supplementary data
Study characteristics	17	Cite each included study and present its characteristics.	Study characteristics (table 1)
Risk of bias in studies	18	Present assessments of risk of bias for each included study.	Supplementary data
Results of individual studies	19	For all outcomes, present, for each study: (a) summary statistics for each group (where appropriate) and (b) an effect estimate and its precision (e.g. confidence/credible interval), ideally using structured tables or plots.	n/a
Results of syntheses	20a	For each synthesis, briefly summarise the characteristics and risk of bias among contributing studies.	Discussion (21-22)
	20b	Present results of all statistical syntheses conducted. If meta-analysis was done, present for each the summary estimate and its precision (e.g. confidence/credible interval) and measures of statistical heterogeneity. If comparing groups, describe the direction of the effect.	n/a
	20c	Present results of all investigations of possible causes of heterogeneity among study results.	Discussion (21-22)
	20d	Present results of all sensitivity analyses conducted to assess the robustness of the synthesized results.	n/a
Reporting biases	21	Present assessments of risk of bias due to missing results (arising from reporting biases) for each synthesis assessed.	Discussion (21-22)

Section and Topic	Item #	Checklist item	Location where item is reported
Certainty of evidence	22	Present assessments of certainty (or confidence) in the body of evidence for each outcome assessed.	n/a
**DISCUSSION**			
Discussion	23a	Provide a general interpretation of the results in the context of other evidence.	Discussion (21-22)
	23b	Discuss any limitations of the evidence included in the review.	Discussion (21- 22)
	23c	Discuss any limitations of the review processes used.	Discussion (21-22)
	23d	Discuss implications of the results for practice, policy, and future research.	Discussion (21- 22)
**OTHER INFORMATION**			
Registration and protocol	24a	Provide registration information for the review, including register name and registration number, or state that the review was not registered.	Design (4)
	24b	Indicate where the review protocol can be accessed, or state that a protocol was not prepared.	Design (4)
	24c	Describe and explain any amendments to information provided at registration or in the protocol.	n/a
Support	25	Describe sources of financial or non-financial support for the review, and the role of the funders or sponsors in the review.	Declaration (24)
Competing interests	26	Declare any competing interests of review authors.	Declaration (24)
Availability of data, code and other materials	27	Report which of the following are publicly available and where they can be found: template data collection forms; data extracted from included studies; data used for all analyses; analytic code; any other materials used in the review.	Have been uploaded to OSF platform

From: Page MJ, McKenzie JE, Bossuyt PM, Boutron I, Hoffmann TC, Mulrow CD, et al. The PRISMA 2020 statement: an updated guideline for reporting systematic reviews. BMJ 2021;372:n71. doi: 10.1136/bmj.n71. This work is licensed under CC BY 4.0. To view a copy of this license, visit https://creativecommons.org/licenses/by/4.0/

Table S2. Study characteristics

Study by first author, year, location	Single or multi-modal intervention	Intervention components	Workshop topics
Briel et al. (2006)(24)<br>Switzerland	Multi-modal	Education workshop<br>Provision of feedback	Patient communication<br>Prescribing guidelines
LeBlanc et al. (2011)(25)<br>Canada	Multi-modal	Education workshop<br>Provision of feedback<br>Decision support tool	Patient communication<br>Involving patients in decision making process<br>Available evidence of risks and benefits in decision to use antibiotics<br>Probabilities of bacterial vs. viral ARIs[1]
Légaré et al. (2012)(26)<br>Canada	Multi-modal	Education workshop<br>Online modules	Patient communication<br>Involving patients in decisions to prescribe antibiotics or not<br>Estimate diagnostic probabilities of ARIs[1]
Lescure et al. (2024)(27)<br>The Netherlands	Multi-modal	Education workshop<br>Online modules<br>Patient materials	Patient communication
Lundborg et al. (1999)(28)<br>Sweden	Single	Education workshop	Feedback on simulated cases<br>Feedback on actual prescribing<br>Peer group discussion<br>Use of guidelines
Magin et al. (2016)(29)<br>Australia	Multi-modal	Education workshop<br>Online modules	Patient communication<br>Prescribing guidelines

Study by first author, year, location	Single or multi-modal intervention	Intervention components	Workshop topics
Magin et al. (2018)(30)<br>Australia	Multi-modal	Education workshop<br>Online modules<br>Separate workshop for supervising GPs	Patient communication<br>Prescribing guidelines<br>Default therapeutic decision should be to not prescribe antibiotics<br>RTIs[2] should be treated syndromically
McNulty et al. (2018)(31)<br>United Kingdom	Multi-modal	Education workshop<br>Patient materials<br>Audit toolkits	Evidence on why antibiotic dispensing is important to control AMR[3]<br>Resources available and how they can be used in practice<br>Discussion of clinical case studies and diagnostic guidance<br>Local antibiotic use data compared with other practices locally and nationally<br>How improved antibiotic use can reduce future consultations<br>How to involve the whole practice in antimicrobial stewardship<br>How practice can take actions forward<br>National antibiotic management practice

Study by first author, year, location	Single or multi-modal intervention	Intervention components	Workshop topics
Morrison et al. (2005)(32) Scotland	Multi-modal	Education workshop<br>Learning needs assessment	Prescribing data<br>Case based discussion
Perez-Cuevas et al. (1996)(33) Mexico	Multi-modal	Education workshop<br>Peer review	Current prescribing rates of participants<br>Appropriate treatment of rhinopharyngitis<br>Discussion of proposed treatment scheme
Petruschke et al. (2021)(34) Germany	Multi-modal	Education workshop<br>GP self-monitoring<br>Digital information prescriptions<br>Printed information prescriptions<br>Posters<br>Waiting room patient leaflet	Development and epidemiology of AMR[3] in Germany<br>Antibiotic use in outpatient care in Germany and Europe<br>Recommendations for antibiotic therapy in Primary Care with an emphasis on ARIs[1]<br>Strategies (including patient communication) for avoiding unnecessary antibiotic prescriptions
Reyes-Morales et al. (2009)(35) Mexico	Multi-modal	Education workshop<br>Training of clinical tutors	Development of clinical guidance for ARIs[1]<br>Prescribing guidelines
Richards et al. (2003)(36) New Zealand	Single	Education workshop	Evidence based use of antibiotics
Rollnick et al. (2002)(37) United Kingdom	Single	Education workshop	Patient communication

Study by first author, year, location	Single or multi-modal intervention	Intervention components	Workshop topics
Sharma et al. (2002)(38)<br>India	Single	Education workshop	Prescribing guidelines<br>Increase in AMR[3]<br>Clinical differentiation of bacterial and viral infections
Smeets et al. (2009)(39)<br>The Netherlands	Single	Education workshop	Patient communication<br>Prescribing guidelines<br>Consensus discussion around indications and 1st choice of antibiotic
Strumann et al. (2020)(40)<br>Germany	Multi-modal	Education workshop<br>Online tool	Patient communication
Welschen et al. (2004)(41)<br>The Netherlands	Multi-modal	Education workshop<br>Provision of feedback<br>Training for GP assistants on patient education and prescribing guidelines<br>Education materials for patients	Patient communication<br>Prescribing guidelines<br>Consensus discussion around indications and 1st choice of antibiotic
Wilf – Miron et al. (2012)(42)<br>Israel	Single	Education workshop	Prescribing data<br>Discussion of factors that impact on groups antibiotic prescribing<br>Barriers and enablers of change

Key: [1] Acute respiratory tract infection
[2]Respiratory tract infection
[3]Antimicrobial resistance

Table S3. MMAT[1] quality evaluation of the studies included in the mixed-method systematic review (n=19)

Study Included, author(s)	Types of mixed methods study components or primary studies	Screening questions and methodological quality criteria	Evaluation			
			Yes	No	Can't tell	Comments
**Briel *et al.* 2006**	**Screening questions**	Are there clear qualitative and quantitative research questions, or a clear mixed methods question?	*			
		Do the collected data allow address the research question (objective)?	*			
	**Quantitative randomized controlled trials**	2.1 Is randomization appropriately performed?	*			
		2.2. Are the groups comparable at baseline?	*			
		2.3. Are there complete outcome data?	*			
		2.4. Are outcome assessors blinded to the intervention provided?			*	
		2.5 Did the participants adhere to the assigned intervention?	*			
**LeBlanc *et al.* 2011**	**Screening questions**	Are there clear qualitative and quantitative research questions, or a clear mixed methods question?	*			
		Do the collected data allow address the research question (objective)?	*			
	**Quantitative randomized controlled trials**	2.1 Is randomization appropriately performed?	*			
		2.2. Are the groups comparable at baseline?	*			
		2.3. Are there complete outcome data?	*			
		2.4. Are outcome assessors blinded to the intervention provided?		*		
		2.5 Did the participants adhere to the assigned intervention?		*		Less than 50% completed full intervention
**Légaré *et al.* 2012**	**Screening questions**	Are there clear qualitative and quantitative research questions, or a clear mixed methods question?	*			
		Do the collected data allow address the research question (objective)?	*			
	**Quantitative randomized controlled trials**	2.1 Is the sampling strategy relevant to address the quantitative research question?	*			
		2.2. Are the groups comparable at baseline?	*			
		2.3. Are there complete outcome data?	*			
		2.4. Are outcome assessors blinded to the intervention provided?			*	
		2.5 Did the participants adhere to the assigned intervention?	*			

Study Included, author(s)	Types of mixed methods study components or primary studies	Screening questions and methodological quality criteria	Evaluation			
			Yes	No	Can't tell	Comments
**Lescure *et al.* 2024**	**Screening questions**	Are there clear qualitative and quantitative research questions, or a clear mixed methods question?	*			
		Do the collected data allow address the research question (objective)?	*			
	**Quantitative non-randomized studies**	3.1. Are the participants representative of the target population?	*			
		3.2. Are measurements appropriate regarding both the outcome and intervention (or exposure)?	*			
		3.3. Are there complete outcome data?	*			
		3.4. Are the confounders accounted for in the design and analysis?	*			
		3.5 During the study period, is the intervention administered (or exposure occurred) as intended?	*			
**Lundborg *et al.* 1999**	**Screening questions**	Are there clear qualitative and quantitative research questions, or a clear mixed methods question?	*			
		Do the collected data allow address the research question (objective)?	*			
	**Quantitative randomized controlled trials**	2.1 Is the sampling strategy relevant to address the quantitative research question?	*			
		2.2. Are the groups comparable at baseline?	*			
		2.3. Are there complete outcome data?	*			
		2.4. Are outcome assessors blinded to the intervention provided?			*	
		2.5 Did the participants adhere to the assigned intervention?	*			
**Magin *et al.* 2016**	**Screening questions**	Are there clear qualitative and quantitative research questions, or a clear mixed methods question?	*			
		Do the collected data allow address the research question (objective)?	*			
	**Quantitative non-randomized studies**	3.1. Are the participants representative of the target population?	*			
		3.2. Are measurements appropriate regarding both the outcome and intervention (or exposure)?	*			
		3.3. Are there complete outcome data?	*			
		3.4. Are the confounders accounted for in the design and analysis?			*	
		3.5 During the study period, is the intervention administered (or exposure occurred) as intended?	*			

Study Included, author(s)	Types of mixed methods study components or primary studies	Screening questions and methodological quality criteria	Evaluation			
			Yes	No	Can't tell	Comments
**Magin *et al.* 2018**	**Screening questions**	Are there clear qualitative and quantitative research questions, or a clear mixed methods question?	*			
		Do the collected data allow address the research question (objective)?	*			
	**Quantitative non-randomized studies**	3.1. Are the participants representative of the target population?	*			
		3.2. Are measurements appropriate regarding both the outcome and intervention (or exposure)?	*			
		3.3. Are there complete outcome data?	*			
		3.4. Are the confounders accounted for in the design and analysis?	*			
		3.5 During the study period, is the intervention administered (or exposure occurred) as intended?	*			
**McNulty *et al.* 2018**	**Screening questions**	Are there clear qualitative and quantitative research questions, or a clear mixed methods question?	*			
		Do the collected data allow address the research question (objective)?	*			
	**Quantitative randomized controlled trials**	2.1 Is the sampling strategy relevant to address the quantitative research question?	*			
		2.2. Are the groups comparable at baseline?	*			
		2.3. Are there complete outcome data?	*			
		2.4. Are outcome assessors blinded to the intervention provided?			*	
		2.5 Did the participants adhere to the assigned intervention?	*			
**Morrison *et al.* 2005**	**Screening questions**	Are there clear qualitative and quantitative research questions, or a clear mixed methods question?	*			
		Do the collected data allow address the research question (objective)?	*			
	**Quantitative non-randomized studies**	3.1. Are the participants representative of the target population?	*			
		3.2. Are measurements appropriate regarding both the outcome and intervention (or exposure)?	*			
		3.3. Are there complete outcome data?	*			
		3.4. Are the confounders accounted for in the design and analysis?			*	
		3.5 During the study period, is the intervention administered (or exposure occurred) as intended?	*			

Study Included, author(s)	Types of mixed methods study components or primary studies	Screening questions and methodological quality criteria	Evaluation			
			**Yes**	**No**	**Can't tell**	**Comments**
**Perez - Cuevas *et al.* 1996**	**Screening questions**	Are there clear qualitative and quantitative research questions, or a clear mixed methods question?	*			
		Do the collected data allow address the research question (objective)?			*	
	**Quantitative non-randomized studies**	3.1. Are the participants representative of the target population?	*			
		3.2. Are measurements appropriate regarding both the outcome and intervention (or exposure)?			*	
		3.3. Are there complete outcome data?		*		
		3.4. Are the confounders accounted for in the design and analysis?			*	
		3.5 During the study period, is the intervention administered (or exposure occurred) as intended?	*			
**Petruschke *et al.* 2021**	**Screening questions**	Are there clear qualitative and quantitative research questions, or a clear mixed methods question?	*			
		Do the collected data allow address the research question (objective)?	*			
	**Quantitative non-randomized studies**	3.1. Are the participants representative of the target population?	*			
		3.2. Are measurements appropriate regarding both the outcome and intervention (or exposure)?	*			
		3.3. Are there complete outcome data?	*			
		3.4. Are the confounders accounted for in the design and analysis?			*	
		3.5 During the study period, is the intervention administered (or exposure occurred) as intended?	*			
**Reyes - Morales *et al.* 2009**	**Screening questions**	Are there clear qualitative and quantitative research questions, or a clear mixed methods question?	*			
		Do the collected data allow address the research question (objective)?	*			
	**Quantitative non-randomized studies**	3.1. Are the participants representative of the target population?	*			
		3.2. Are measurements appropriate regarding both the outcome and intervention (or exposure)?	*			
		3.3. Are there complete outcome data?	*			
		3.4. Are the confounders accounted for in the design and analysis?			*	
		3.5 During the study period, is the intervention administered (or exposure occurred) as intended?	*			

Study Included, author(s)	Types of mixed methods study components or primary studies	Screening questions and methodological quality criteria	Evaluation			
			Yes	No	Can't tell	Comments
**Richards *et al.* 2003**	**Screening questions**	Are there clear qualitative and quantitative research questions, or a clear mixed methods question?	*			
		Do the collected data allow address the research question (objective)?	*			
	**Quantitative non-randomized studies**	3.1. Are the participants representative of the target population?	*			
		3.2. Are measurements appropriate regarding both the outcome and intervention (or exposure)?	*			
		3.3. Are there complete outcome data?	*			
		3.4. Are the confounders accounted for in the design and analysis?	*			
		3.5 During the study period, is the intervention administered (or exposure occurred) as intended?	*			
**Rollnick *et al.* 2002**	**Screening questions**	Are there clear qualitative and quantitative research questions, or a clear mixed methods question?		*		
		Do the collected data allow address the research question (objective)?			*	
	**Qualitative studies**	1.1. Is the qualitative approach appropriate to answer the research question?	*			
		1.2. Are the qualitative data collection methods adequate to address the research question?	*			
		1.3. Are the findings adequately derived from the data?			*	
		1.4. Is the interpretation of results sufficiently substantiated by data?		*		
		1.5. Is there coherence between qualitative data sources, collection, analysis and interpretation?	*			
**Sharma *et al.* 2002**	**Screening questions**	Are there clear qualitative and quantitative research questions, or a clear mixed methods question?	*			
		Do the collected data allow address the research question (objective)?	*			
	**Quantitative randomized controlled trials**	2.1 Is randomization appropriately performed?			*	
		2.2. Are the groups comparable at baseline?	*			
		2.3. Are there complete outcome data?	*			
		2.4. Are outcome assessors blinded to the intervention provided?			*	
		2.5 Did the participants adhere to the assigned intervention?	*			

Study Included, author(s)	Types of mixed methods study components or primary studies	Screening questions and methodological quality criteria	Evaluation			
			Yes	No	Can't tell	Comments
**Smeets *et al.* 2009**	**Screening questions**	Are there clear qualitative and quantitative research questions, or a clear mixed methods question?	*			
		Do the collected data allow address the research question (objective)?	*			
	**Quantitative non-randomized studies**	3.1. Are the participants representative of the target population?	*			
		3.2. Are measurements appropriate regarding both the outcome and intervention (or exposure)?	*			
		3.3. Are there complete outcome data?	*			
		3.4. Are the confounders accounted for in the design and analysis?	*			
		3.5 During the study period, is the intervention administered (or exposure occurred) as intended?	*			
**Strumann *et al.* 2020**	**Screening questions**	Are there clear qualitative and quantitative research questions, or a clear mixed methods question?	*			
		Do the collected data allow address the research question (objective)?	*			
	**Quantitative non-randomized studies**	3.1. Are the participants representative of the target population?	*			
		3.2. Are measurements appropriate regarding both the outcome and intervention (or exposure)?	*			
		3.3. Are there complete outcome data?	*			
		3.4. Are the confounders accounted for in the design and analysis?			*	
		3.5 During the study period, is the intervention administered (or exposure occurred) as intended?	*			
**Welschen *et al.* 2004**	**Screening questions**	Are there clear qualitative and quantitative research questions, or a clear mixed methods question?	*			
		Do the collected data allow address the research question (objective)?	*			
	**Quantitative randomized controlled trials**	2.1 Is the sampling strategy relevant to address the quantitative research question?	*			
		2.2. Are the groups comparable at baseline?	*			
		2.3. Are there complete outcome data?	*			
		2.4. Are outcome assessors blinded to the intervention provided?			*	
		2.5 Did the participants adhere to the assigned intervention?	*			

Study Included, author(s)	Types of mixed methods study components or primary studies	Screening questions and methodological quality criteria	Evaluation			
			Yes	No	Can't tell	Comments
**Wilf - Miron *et al.* 2012**	**Screening questions**	Are there clear qualitative and quantitative research questions, or a clear mixed methods question?	*			
		Do the collected data allow address the research question (objective)?	*			
	**Quantitative non-randomized studies**	3.1 Are the participants representative of the target population?	*			
		3.2. Are measurements appropriate regarding both the outcome and intervention (or exposure)?	*			
		3.3. Are there complete outcome data?	*			
		3.4. Are the confounders accounted for in the design and analysis?			*	
		3.5 During the study period, is the intervention administered (or exposure occurred) as intended?	*			

[1] MMAT – Mixed Methods Appraisal Tool

Hong QN, Pluye P, Fàbregues S, Bartlett G, Boardman F, Cargo M, Dagenais P, Gagnon M-P, Griffiths F, Nicolau B, O'Cathain A, Rousseau M-C, Vedel I.

Mixed Methods Appraisal Tool (MMAT), version 2018. Registration of Copyright (#1148552), Canadian Intellectual Property Office, Industry Canada.

Excluded studies

1. Allaire AS, Labrecque M, Giguere A, Gagnon MP, Grimshaw J, Legare F. Barriers and facilitators to the dissemination of DECISION+, a continuing medical education program for optimizing decisions about antibiotics for acute respiratory infections in primary care: a study protocol. Implementation science : IS. 2011;6(101258411):3.

2. Allison R, Lecky DM, Beech E, Ashiru-Oredope D, Costelloe C, Owens R, et al. What Resources do NHS Commissioning Organisations Use to Support Antimicrobial Stewardship in Primary Care in England?. Antibiotics (Basel, Switzerland). 2020;9(4).

3. Altiner A, Berner R, Diener A, Feldmeier G, Köchling A, Löffler C, et al. Converting habits of antibiotic prescribing for respiratory tract infections in German primary care--the cluster-randomized controlled CHANGE-2 trial. BMC family practice. 2012;13((Altiner A.) Institute of General Practice, Rostock University Medical Center, Rostock, Germany.(Berner R.; Diener A.; Feldmeier G.; Köchling A.; Löffler C.; Schröder H.; Siegel A.; Wollny A.; Kern W.V.)):124.

4. Altiner A, Brockmann S, Sielk M, Wilm S, Wegscheider K, Abholz HH. Reducing antibiotic prescriptions for acute cough by motivating GPs to change their attitudes to communication and empowering patients: a cluster-randomized intervention study. The Journal of antimicrobial chemotherapy. 2007;60(3):638–44.

5. Alves PG, Hayward G, Leydon G, Barnes R, Woods C, Webb J, et al. Antibiotic prescribing in UK out-of-hours primary care services: a realist-informed scoping review of training and guidelines for healthcare professionals. BJGP Open. 2021;5(3):1–9.

6. Anthierens S, Tonkin-Crine S, Cals JW, Coenen S, Yardley L, Brookes-Howell L, et al. Clinicians' views and experiences of interventions to enhance the quality of antibiotic prescribing for acute respiratory tract infections. Journal of general internal medicine. 2015;30(4):408–16.

7. Arnold SR, Straus SE. Interventions to improve antibiotic prescribing practices in ambulatory care. The Cochrane database of systematic reviews. 2005;(4):CD003539.

8. Arnold SH, Olesen JA, Jensen JN, Bjerrum L, Holm A, Kousgaard MB. Development of a tailored, complex intervention for clinical reflection and communication about suspected urinary tract infections in nursing home residents. Antibiotics. 2020;9(6):1–16.

9. Asai Y, Konishi T, Yamamoto T, Chikazawa K, Nakano M, Kinoshita E, et al. Impact of antimicrobial stewardship program-driven educational intervention for vancomycin loading dose on mortality. Journal of Infection and Chemotherapy. 2023;29(11):1023–32.

10. Avdic E, Cushinotto LA, Hughes AH, Hansen AR, Efird LE, Bartlett JG, et al. Impact of an antimicrobial stewardship intervention on shortening the duration of therapy for community-acquired pneumonia. Clinical Infectious Diseases. 2012;54(11):1581–7.

11. Bekkers MJ, Simpson SA, Dunstan F, Hood K, Hare M, Evans J, et al. Enhancing the quality of antibiotic prescribing in primary care: qualitative evaluation of a blended learning intervention. BMC family practice. 2010;11(100967792):34.

12. Belongia EA, Sullivan BJ, Chyou PH, Madagame E, Reed KD, Schwartz B. A community intervention trial to promote judicious antibiotic use and reduce penicillin-resistant Streptococcus pneumoniae carriage in children. Pediatrics. 2001;108(3):575–83.

13. Bjerrum L, Munck A, Gahrn-Hansen B, Hansen MP, Jarboel D, Llor C, et al. Health Alliance for Prudent Prescribing, Yield and Use of Antimicrobial Drugs in the Treatment of Respiratory Tract Infections (HAPPY AUDIT). BMC Family Practice. 2010;11:7p–7p.

14. Bosisio V, Stella R, Castagna S, Marchetti F. A prospective, descriptive study on the practice of management of urinary tract infections by general pratictioners in Italy: Clinical pathways (Part II). Archivio Italiano di Urologia e Andrologia. 2003;75(2):93–8.

15. Brown CA. Reducing Outpatient Antibiotic Prescribing for Acute Respiratory Infections: A Quasi-Experimental Study. Journal of Doctoral Nursing Practice. 2018;11(1):3–15.

16. Butler CC, Simpson SA, Dunstan F, Rollnick S, Cohen D, Gillespie D, et al. Effectiveness of multifaceted educational programme to reduce antibiotic dispensing in primary care: practice based randomised controlled trial. BMJ (Clinical research ed). 2012;344(8900488, bmj, 101090866):d8173.

17. Cals JWL, Butler CC, Hopstaken RM, Hood K, Dinant GJ. Effect of point of care testing for C reactive protein and training in communication skills on antibiotic use in lower respiratory tract infections: cluster randomised trial. BMJ (Clinical research ed). 2009;338(8900488, bmj, 101090866):b1374.

18. Chang Y, Cui Z, He X, Zhou X, Zhou H, Fan X, et al. Effect of unifaceted and multifaceted interventions on antibiotic prescription control for respiratory diseases: A systematic review of randomized controlled trials. Medicine (United States). 2022;101(41):E30865.

19. Chazan B, Turjeman RBZ, Frost Y, Besharat B, Tabenkin H, Stainberg A, et al. Antibiotic consumption successfully reduced by a community intervention program. Israel Medical Association Journal. 2007;9(1):16–20.

20. Davidson LE, Gentry EM, Priem JS, Kowalkowski M, Spencer MD. A multimodal intervention to decrease inappropriate outpatient antibiotic prescribing for upper respiratory tract infections in a large integrated healthcare system. Infection Control and Hospital Epidemiology. 2023;44(3):392–9.

21. De Santis G, Harvey KJ, Howard D, Mashford ML, Moulds RF. Improving the quality of antibiotic prescription patterns in general practice. The role of educational intervention. The Medical journal of Australia. 1994;160(8):502–5.

22. Delsors E, Monsó F, López-Román FJ, Menárguez-Puche JF, Gonzalez-Barberá M, Hukelova H, et al. Changes in antibiotic prescription following an education strategy for acute respiratory infections. npj Primary Care Respiratory Medicine [Internet]. 2021;31(1). Available from: https://www.embase.com/search/results?subaction=viewrecord&id=L2012311256&from=export

23. Dollman WB, LeBlanc VT, Stevens L, O'Connor PJ, Turnidge JD. A community-based intervention to reduce antibiotic use for upper respiratory tract infections in regional South Australia. Medical Journal of Australia. 2005;182(12):617–20.

24. Duane S, Callan A, Galvin S, Murphy AW, Domegan C, O'Shea E, et al. Supporting the improvement and management of prescribing for urinary tract infections (SIMPle): protocol for a cluster randomized trial. Trials. 2013;14(101263253):441.

25. Dutcher L, Degnan K, Adu-Gyamfi AB, Lautenbach E, Cressman L, David MZ, et al. Improving Outpatient Antibiotic Prescribing for Respiratory Tract Infections in Primary Care: A Stepped-Wedge Cluster Randomized Trial. Clinical Infectious Diseases. 2022;74(6):947–56.

26. Enriquez-Puga A, Baker R, Paul S, Villoro-Valdes R. Effect of educational outreach on general practice prescribing of antibiotics and antidepressants: A two-year randomised controlled trialal. Scandinavian Journal of Primary Health Care. 2009;27(4):195–201.

27. Enriquez-Puga A, Baker R, Paul S, Villoro-Valdes R. Effect of educational outreach on general practice prescribing of antibiotics and antidepressants: a two-year randomised controlled trial. Scandinavian journal of primary health care. 2009;27(4):195–201.

28. Esmaily HM, Savage C, Vahidi R, Amini A, Dastgiri S, Hult H, et al. Does an outcome-based approach to continuing medical education improve physicians' competences in rational prescribing? Medical Teacher. 2009;31(11):e500–6.

29. Esmaily HM, Silver I, Shiva S, Gargani A, Maleki-Dizaji N, Al-Maniri A, et al. Can rational prescribing be improved by an outcome-based educational approach? A randomized trial completed in Iran. The Journal of continuing education in the health professions. 2010;30(1):11–8.

30. Fernandez Urrusuno R, Flores Dorado M, Vilches Arenas A, Serrano Martino C, Corral Baena S, Montero Balosa MC. Improving the appropriateness of antimicrobial use in primary care after implementation of a local antimicrobial guide in both levels of care. European journal of clinical pharmacology. 2014;70(8):1011–20.

31. Ferrat E, Le Breton J, Guery E, Adeline F, Audureau E, Montagne O, et al. Effects 4.5 years after an interactive GP educational seminar on antibiotic therapy for respiratory tract infections: a randomized controlled trial. Family practice. 2016;33(2):192–9.

32. Figueiras A, López-Vázquez P, Gonzalez-Gonzalez C, Vázquez-Lago JM, Piñeiro-Lamas M, López-Durán A, et al. Impact of a multifaceted intervention to improve antibiotic prescribing: a pragmatic cluster-randomised controlled trial. Antimicrobial Resistance and Infection Control [Internet]. 2020;9(1). Available from: https://www.embase.com/search/results?subaction=viewrecord&id=L2007542711&from=export

33. Finkelstein JA, Davis RL, Dowell SF, Metlay JP, Soumerai SB, Rifas-Shiman SL, et al. Reducing antibiotic use in children: a randomized trial in 12 practices. Pediatrics. 2001;108(1):1–7.

34. Finkelstein JA, Huang SS, Kleinman K, Rifas-Shiman SL, Stille CJ, Daniel J, et al. Impact of a 16-community trial to promote judicious antibiotic use in Massachusetts. Pediatrics. 2008;121(1):e15-23.

35. Font M, Madridejos R, Catalan A, Jimenez J, Argimon JM, Huguet M. [Improving drug prescription in primary care: a controlled and randomized study of an educational method]. Medicina clinica. 1991;96(6):201–5.

36. Frich JC, Hoye S, Lindbaek M, Straand J. General practitioners and tutors' experiences with peer group academic detailing: a qualitative study. BMC family practice. 2010;11(100967792):12.

37. Garjani A, Salimnejad M, Shamsmohamadi M, Baghchevan V, Vahidi RG, Maleki-Dijazi N, et al. Effect of interactive group discussion among physicians to promote rational prescribing. Eastern Mediterranean health journal = La revue de sante de la Mediterranee orientale = al-Majallah al-sihhiyah li-sharq al-mutawassit. 2009;15(2):408–15.

38. Germeni E, Frost J, Garside R, Rogers M, Valderas JM, Britten N. Antibiotic prescribing for acute respiratory tract infections in primary care: An updated and expanded meta-ethnography. British Journal of General Practice. 2018;68(674):e633–45.

39. Gjelstad S, Fetveit A, Straand J, Dalen I, Rognstad S, Lindbaek M. Can antibiotic prescriptions in respiratory tract infections be improved? A cluster-randomized educational intervention in general practice--the Prescription Peer Academic Detailing (Rx-PAD) Study [NCT00272155]. BMC health services research. 2006;6(101088677):75.

40. Gjelstad S, Hoye S, Straand J, Brekke M, Dalen I, Lindbaek M. Improving antibiotic prescribing in acute respiratory tract infections: cluster randomised trial from Norwegian general practice (prescription peer academic detailing (Rx-PAD) study). BMJ (Clinical research ed). 2013;347(8900488, bmj, 101090866):f4403.

41. Gonzales R, Steiner JF, Lum A, Barrett PHJ. Decreasing antibiotic use in ambulatory practice: impact of a multidimensional intervention on the treatment of uncomplicated acute bronchitis in adults. JAMA. 1999;281(16):1512–9.

42. Gornyk D, Scharlach M, Buhr-Riehm B, Klett-Tammen CJ, Eberhard S, Stahmeyer JT, et al. Effectiveness of Trainings of General Practitioners on Antibiotic Stewardship: Methods of a Pragmatic Quasi-Experimental Study in a Controlled Before-After Design in South-East-Lower Saxony, Germany (WASA). Frontiers in Pharmacology [Internet]. 2021;12((Gornyk D.; Klett-Tammen C.J.; Krause G.; Castell S., Stefanie.Castell@helmholtz-hzi.de) Department of Epidemiology, Helmholtz Centre for Infection Research, Braunschweig, Germany(Gornyk D.) PhD Programme Epidemiology Hannover-Braunschweig, Braunschweig,). Available from: https://www.embase.com/search/results?subaction=viewrecord&id=L634937669&from=export

43. Greene RA, Beckman H, Chamberlain J, Partridge G, Miller M, Burden D, et al. Increasing adherence to a community-based guideline for acute sinusitis through education, physician profiling, and financial incentives. American Journal of Managed Care. 2004;10(10):670–8.

44. Gutierrez G, Guiscafre H, Bronfman M, Walsh J, Martinez H, Munoz O. Changing physician prescribing patterns: evaluation of an educational strategy for acute diarrhea in Mexico City. Medical care. 1994;32(5):436–46.

45. Harrigan JJ, Hamilton KW, Cressman L, Bilker WB, Degnan KO, David MZ, et al. Antibiotic Prescribing Patterns for Respiratory Tract Illnesses Following the Conclusion of an Education and Feedback Intervention in Primary Care. Clinical infectious diseases : an official publication of the Infectious Diseases Society of America [Internet]. 2024;((Harrigan J.J.; Hamilton K.W.; Degnan K.O.; David M.Z.; Pegues D.A.; Dutcher L.) Division of Infectious Diseases, Department of Medicine, University of Pennsylvania Perelman School of Medicine, Philadelphia, PA, United States(Cressman L.; Bilker W.B.) Dep). Available from: https://www.embase.com/search/results?subaction=viewrecord&id=L643342439&from=export

46. Hoye S, Gjelstad S, Lindbaek M. Effects on antibiotic dispensing rates of interventions to promote delayed prescribing for respiratory tract infections in primary care. The British journal of general practice : the journal of the Royal College of General Practitioners. 2013;63(616):e777-86.

47. Ivanovska V, Holloway KA. Interventions to improve antibiotic prescribing in upper middle income countries: A systematic review of the literature 1990-2009. Macedonian Journal of Medical Sciences. 2013;6(1):84–91.

48. Ives TJ, Frey JJ, Furr SJ, Bentz EJ. Effect of an educational intervention on oral cephalosporin use in primary care. Archives of internal medicine. 1987;147(1):44–7.

49. Juzych NS, Banerjee M, Essenmacher L, Lerner SA. Improvements in antimicrobial prescribing for treatment of upper respiratory tract infections through provider education. Journal of general internal medicine. 2005;20(10):901–5.

50. Kandeel A, Palms DL, Afifi S, Kandeel Y, Etman A, Hicks LA, et al. An educational intervention to promote appropriate antibiotic use for acute respiratory infections in a district in Egypt- pilot study. BMC public health. 2019;19(Suppl 3):498.

51. Katz MJ, Gurses AP, Tamma PD, Cosgrove SE, Miller MA, Jump RLP. Implementing Antimicrobial Stewardship in Long-term Care Settings: An Integrative Review Using a Human Factors Approach. Clinical Infectious Diseases. 2017;65(11):1943–51.

52. Kelley M, Massing MW, Young J, Rogers A, Taylor R, Weiser R. Feasibility of a primary care intervention to decrease oral antibiotics for acute upper respiratory tract infections: A pilot study. North Carolina medical journal. 2006;67(4):249–54.

53. Kiang KM, Kieke BA, Como-Sabetti K, Lynfield R, Besser RE, Belongia EA. Clinician knowledge and beliefs after statewide program to promote appropriate antimicrobial drug use. Emerging infectious diseases. 2005;11(6):904–11.

54. Klein LE, Charache P, Johannes RS. Effect of physician tutorials on prescribing patterns of graduate physicians. Journal of medical education. 1981;56(6):504–11.

55. Korom RR, Onguka S, Halestrap P, McAlhaney M, Adam M. Brief educational interventions to improve performance on novel quality metrics in ambulatory settings in Kenya: A multi-site pre-post effectiveness trial. PloS one. 2017;12(4):e0174566.

56. Kotwani A, Wattal C, Katewa S, Joshic PC, Holloway K. Factors influencing primary care physicians to prescribe antibiotics in Delhi India. Family Practice. 2010;27(6):684–90.

57. Lalana-Josa P, Laclaustra-Mendizábal B, Aza-Pascual-Salcedo MM, Carcas-De-Benavides C, Lallana-Álvarez MJ, Pina-Gadea MB. Does the prescribing of antibiotics in paediatrics improve after a multidisciplinary intervention? Enfermedades Infecciosas y Microbiologia Clinica. 2015;33(2):78–83.

58. Lampi E, Carlsson F, Sundvall PD, Torres MJ, Ulleryd P, Åhrén C, et al. Interventions for prudent antibiotic use in primary healthcare: an econometric analysis. BMC Health Services Research. 2020;20(1):N.PAG-N.PAG.

59. Last K, Simon A, Gärtner BC, Becker SL, Papan C. Attitudes of primary care physicians towards antimicrobial stewardship and the impact of a multi-part training course – a pilot study. GMS Hygiene & Infection Control. 2023;18:1–9.

60. Le Corvoisier P, Renard V, Roudot-Thoraval F, Cazalens T, Veerabudun K, Canoui-Poitrine F, et al. Long-term effects of an educational seminar on antibiotic prescribing by GPs: a randomised controlled trial. The British journal of general practice : the journal of the Royal College of General Practitioners. 2013;63(612):e455-64.

61. Likopa Z, Kivite-Urtane A, Silina V, Pavare J. Impact of educational training and C-reactive protein point-of-care testing on antibiotic prescribing in rural and urban family physician practices in Latvia: a randomised controlled intervention study. BMC Pediatrics [Internet]. 2022;22(1). Available from: https://www.embase.com/search/results?subaction=viewrecord&id=L2019167994&from=export

62. Llor C, Cots JM, López-Valcárcel BG, Arranz J, García G, Ortega J, et al. Interventions to reduce antibiotic prescription for lower respiratory tract infections: Happy Audit study. European Respiratory Journal. 2012;40(2):436–41.

63. Llor C, Bjerrum L, Molero JM, Moragas A, Gonzalez Lopez-Valcarcel B, Monedero MJ, et al. Long-term effect of a practice-based intervention (HAPPY AUDIT) aimed at reducing antibiotic prescribing in patients with respiratory tract infections. The Journal of antimicrobial chemotherapy. 2018;73(8):2215–22.

64. Llor C, Monedero MJ, Garcia G, Arranz J, Cots JM, Bjerrum L. Interventions to improve adherence to first-line antibiotics in respiratory tract infections. The impact depends on the intensity of the intervention. The European journal of general practice. 2015;21(1):12–8.

65. Madridejos-Mora R, Amado-Guirado E, Perez-Rodriguez MT. Effectiveness of the combination of feedback and educational recommendations for improving drug prescription in general practice. Medical care. 2004;42(7):643–8.

66. Magin PJ, Morgan S, Tapley A, Henderson KM, Holliday EG, Ball J, et al. Changes in early-career family physicians' antibiotic prescribing for upper respiratory tract infection and acute bronchitis: A multicentre longitudinal study. Family Practice. 2016;33(4):360–7.

67. Magrini N, Formoso G, Capelli O, Maestri E, Nonino F, Paltrinieri B, et al. Long term effectiveness on prescribing of two multifaceted educational interventions: Results of two large scale randomized cluster trials. PLoS ONE [Internet]. 2014;9(10). Available from: https://www.embase.com/search/results?subaction=viewrecord&id=L600182946&from=export

68. Mbonye MK, Burnett SM, Burua A, Colebunders R, Crozier I, Kinoti SN, et al. Effect of integrated capacity-building interventions on malaria case management by health professionals in Uganda: A mixed design study with pre/post and cluster randomized trial components. PLoS ONE [Internet]. 2014;9(1). Available from: https://www.embase.com/search/results?subaction=viewrecord&id=L372718716&from=export

69. McDonagh MS, Peterson K, Winthrop K, Cantor A, Lazur BH, Buckley DI. Interventions to reduce inappropriate prescribing of antibiotics for acute respiratory tract infections: summary and update of a systematic review. The Journal of international medical research. 2018;46(8):3337–57.

70. McIsaac WJ, Senthinathan A, Moineddin R, Nakamachi Y, Dresser L, McIntyre M, et al. Development and evaluation of a primary care antimicrobial stewardship program (PC-ASP) in Toronto, Ontario, Canada. JAMMI. 2021;6(1):32–48.

71. McKay RM, Vrbova L, Fuertes E, Chong M, David S, Dreher K, et al. Evaluation of the do bugs need drugs? Program in British Columbia: Can we curb antibiotic prescribing? Canadian Journal of Infectious Diseases and Medical Microbiology. 2011;22(1):19–24.

72. McNulty CA, Kane A, Foy CJ, Sykes J, Saunders P, Cartwright KA. Primary care workshops can reduce and rationalize antibiotic prescribing. The Journal of antimicrobial chemotherapy. 2000;46(3):493–9.

73. McNulty CAM, Francis NA. Optimizing antibiotic prescribing in primary care settings in the UK: findings of a BSAC multi-disciplinary workshop 2009. The Journal of antimicrobial chemotherapy. 2010;65(11):2278–84.

74. Mohagheghi MA, Mosavi-Jarrahi A, Khatemi-Moghaddam M, Afhami S, Khodai S, Azemoodeh O. Community-based outpatient practice of antibiotics use in Tehran. Pharmacoepidemiology and drug safety. 2005;14(2):135–8.

75. Molero JM, Moragas A, Gonzalez Lopez-Valcarcel B, Bjerrum L, Cots JM, Llor C. Reducing antibiotic prescribing for lower respiratory tract infections 6 years after a multifaceted intervention. International journal of clinical practice. 2019;73(5):e13312.
76. Molstad S, Ekedahl A, Hovelius B, Thimansson H. Antibiotic prescription in primary care: A 5-year follow-up of an educational programme. Family Practice. 1994;11(3):282–6.

77. Monette J, Miller MA, Monette M, Laurier C, Boivin JF, Sourial N, et al. Effect of an educational intervention on optimizing antibiotic prescribing in long-term care facilities. Journal of the American Geriatrics Society. 2007;55(8):1231–5.

78. Neels AJ, Bloch AE, Gwini SM, Athan E. The effectiveness of a simple antimicrobial stewardship intervention in general practice in Australia: A pilot study. BMC Infectious Diseases [Internet]. 2020;20(1). Available from: https://www.embase.com/search/results?subaction=viewrecord&id=L632556755&from=export

79. Oesterle J, Sternemann M, Sande T, Aplin-Kalisz C, Towers D. Antimicrobial Resistance Education in the Primary Care Setting. Journal of Doctoral Nursing Practice. 2016;9(2):217–25.

80. Onion CW, Bartzokas CA. Changing attitudes to infection management in primary care: a controlled trial of active versus passive guideline implementation strategies. Family practice. 1998;15(2):99–104.

81. Pasay DK, Guirguis MS, Shkrobot RC, Slobodan JP, Wagg AS, Sadowski CA, et al. Antimicrobial stewardship in rural nursing homes: Impact of interprofessional education and clinical decision tool implementation on urinary tract infection treatment in a cluster randomized trial. Infection control and hospital epidemiology. 2019;40(4):432–7.

82. Peñalva G, Crespo-Rivas JC, Guisado-Gil AB, Rodríguez-Villodres Á, Pachón-Ibáñez ME, Cachero-Alba B, et al. Clinical and Ecological Impact of an Educational Program to Optimize Antibiotic Treatments in Nursing Homes (PROA-SENIOR): A Cluster, Randomized, Controlled Trial and Interrupted Time-Series Analysis. Clinical Infectious Diseases. 2023;76(5):824–32.

83. Peñalva G, Fernández-Urrusuno R, Turmo JM, Hernández-Soto R, Pajares I, Carrión L, et al. Long-term impact of an educational antimicrobial stewardship programme in primary care on infections caused by extended-spectrum β-lactamase-producing Escherichia coli in the community: an interrupted time-series analysis. The Lancet Infectious Diseases. 2020;20(2):199–207.

84. Persell SD, Doctor JN, Friedberg MW, Meeker D, Friesema E, Cooper A, et al. Behavioral interventions to reduce inappropriate antibiotic prescribing: a randomized pilot trial. BMC infectious diseases. 2016;16(100968551):373.

85. Perz JF, Craig AS, Coffey CS, Jorgensen DM, Mitchel E, Hall S, et al. Changes in antibiotic prescribing for children after a community-wide campaign. JAMA: Journal of the American Medical Association. 2002;287(23):3103–54.

86. Pettersson E, Vernby Å, lstad S, Lundborg CS. Can a multifaceted educational intervention targeting both nurses and physicians change the prescribing of antibiotics to nursing home residents? A cluster randomized controlled trial. Journal of Antimicrobial Chemotherapy. 2011;66(11):2659–66.

87. Pichichero ME. Preferred antibiotics for treatment of acute otitis media: comparison of practicing pediatricians, general practitioners, and otolaryngologists. Clinical pediatrics. 2005;44(7):575–8.

88. Plachouras D, Antoniadou A, Giannitsioti E, Galani L, Katsarolis I, Kavatha D, et al. Promoting prudent use of antibiotics: the experience from a multifaceted regional campaign in Greece. BMC public health. 2014;14(100968562):866.

89. Pos-Doering R, Kuehn L, Kamradt M, Glassen K, Fleischhauer T, Kaufmann-Kolle P, et al. Converting habits of antibiotic use for respiratory tract infections in German primary care (CHANGE-3) - process evaluation of a complex intervention. BMC family practice. 2020;21(1):274.

90. Potena A, Simoni M, Cellini M, Cartabellotta A, Ballerin L, Piattella M, et al. Management of community-acquired pneumonia by trained family general practitioners. International Journal of Tuberculosis and Lung Disease. 2008;12(1):19–25.

91. Razon Y, Ashkenazi S, Cohen A, Hering E, Amzel S, Babilsky H, et al. Effect of educational intervention on antibiotic prescription practices for upper respiratory infections in children: A multicentre study. Journal of Antimicrobial Chemotherapy. 2005;56(5):937–40.

92. Regev-Yochay G, Raz M, Dagan R, Roizin H, Morag B, Hetman S, et al. Reduction in antibiotic use following a cluster randomized controlled multifaceted intervention: The Israeli judicious antibiotic prescription study. Clinical Infectious Diseases. 2011;53(1):33–41.

93. Rocha V, Estrela M, Neto V, Roque F, Figueiras A, Herdeiro MT. Educational Interventions to Reduce Prescription and Dispensing of Antibiotics in Primary Care: A Systematic Review of Economic Impact. Antibiotics [Internet]. 2022;11(9). Available from: https://www.embase.com/search/results?subaction=viewrecord&id=L2019186669&from=export

94. Roque F, Herdeiro MT, Soares S, Teixeira Rodrigues A, Breitenfeld L, Figueiras A. Educational interventions to improve prescription and dispensing of antibiotics: a systematic review. BMC public health. 2014;14(100968562):1276.

95. Roque F, Teixeira-Rodrigues A, Breitenfeld L, Pineiro-Lamas M, Figueiras A, Herdeiro MT. Decreasing antibiotic use through a joint intervention targeting physicians and pharmacists. Future microbiology. 2016;11(101278120):877–86.

96. Rowe TA, Linder JA. Novel approaches to decrease inappropriate ambulatory antibiotic use. Expert Review of Anti-Infective Therapy. 2019;17(7):511–21.

97. Sangwan R, Neels AJ, Gwini SM, Saha SK, Athan E. Is Education Alone Enough to Sustain Improvements of Antimicrobial Stewardship in General Practice in Australia? Results of an Intervention Follow-Up Study. Antibiotics [Internet]. 2023;12(3). Available from: https://www.embase.com/search/results?subaction=viewrecord&id=L2022294734&from=export

98. Satterfield J, Miesner AR, Percival KM. The role of education in antimicrobial stewardship. The Journal of hospital infection. 2020;105(2):130–41.

99. Schaffner W, Ray WA, Federspiel CF, Miller WO. Improving antibiotic prescribing in office practice. A controlled trial of three educational methods. Journal of the American Medical Association. 1983;250(13):1728–32.

100. Stille CJ, Rifas-Shiman SL, Kleinman K, Kotch JB, Finkelstein JA. Physician responses to a community-level trial promoting judicious antibiotic use. Annals of family medicine. 2008;6(3):206–12.

101. Temte JL, Shult PA, Kirk CJ, Amspaugh J. Effects of viral respiratory disease education and surveillance on antibiotic prescribing. Family medicine. 1999;31(2):101–6.

102. van der Velden AW, Kuyvenhoven MM, Verheij TJM. Improving antibiotic prescribing quality by an intervention embedded in the primary care practice accreditation: The ARTI4 randomized trial. Journal of Antimicrobial Chemotherapy. 2016;71(1):257–63.

103. van der Velden AW, Pijpers EJ, Kuyvenhoven MM, Tonkin-Crine SKG, Little P, Verheij TJM. Effectiveness of physician-targeted interventions to improve antibiotic use for respiratory tract infections. The British journal of general practice : the journal of the Royal College of General Practitioners. 2012;62(605):e801-7.

104. Vervloet M, Meulepas MA, Cals JWL, Eimers M, van der Hoek LS, van Dijk L. Reducing antibiotic prescriptions for respiratory tract infections in family practice: results of a cluster randomized controlled trial evaluating a multifaceted peer-group-based intervention. NPJ primary care respiratory medicine. 2016;26(101631999):15083.

105. Wei X, Zhang Z, Hicks JP, Walley JD, King R, Newell JN, et al. Long-term outcomes of an educational intervention to reduce antibiotic prescribing for childhood upper respiratory tract infections in rural China: Follow-up of a cluster-randomised controlled trial. PLoS Medicine [Internet]. 2019;16(2). Available from: https://www.embase.com/search/results?subaction=viewrecord&id=L2001605582&from=export

106. Wei X, Zhang Z, Walley JD, Hicks JP, Zeng J, Deng S, et al. Effect of a training and educational intervention for physicians and caregivers on antibiotic prescribing for upper respiratory tract infections in children at primary care facilities in rural China: a cluster-randomised controlled trial. The Lancet Global Health. 2017;5(12):e1258–67.

107. Wharton A, Jerome-D'Emilia B, Avallone M. Improving Antibiotic Overuse in Primary Care: A Multimodal Quality Improvement Project. Clinical Nurse Specialist: The Journal for Advanced Nursing Practice. 2024;38(3):136–40.

108. Wong G, Brennan N, Mattick K, Pearson M, Briscoe S, Papoutsi C. Interventions to improve antimicrobial prescribing of doctors in training: the IMPACT (IMProving Antimicrobial presCribing of doctors in Training) realist review. BMJ open. 2015;5(10):e009059.

109. Zabarsky TF, Sethi AK, Donskey CJ. Sustained reduction in inappropriate treatment of asymptomatic bacteriuria in a long-term care facility through an educational intervention. American journal of infection control. 2008;36(7):476–80.

110. Zwar N, Wolk J, Gordon J, Sanson-Fisher R, Kehoe L. Influencing antibiotic prescribing in general practice: a trial of prescriber feedback and management guidelines. Family practice. 1999;16(5):495–500.
